# Incidence of benign and malignant peri‐implant fluid collections and masses on magnetic resonance imaging in women with silicone implants

**DOI:** 10.1002/cam4.2189

**Published:** 2019-09-30

**Authors:** Elizabeth J. Sutton, Brittany Z. Dashevsky, Elizabeth J. Watson, Neelam Tyagi, Blanca Bernard‐Davila, Danny Martinez, Ahmet Dogan, Steven M. Horwitz, Peter G. Cordeiro, Elizabeth A. Morris

**Affiliations:** ^1^ Department of Radiology Memorial Sloan Kettering Cancer Center New York New York; ^2^ Department of Radiology University of California San Francisco California; ^3^ Department of Radiology University of Vermont Burlington Vermont; ^4^ Department of Medical Physics Memorial Sloan Kettering Cancer Center New York New York; ^5^ Department of Pathology and Laboratory Medicine Memorial Sloan Kettering Cancer Center New York New York; ^6^ Departmemt of Medicine Memorial Sloan Kettering Cancer Center New York New York; ^7^ Department of Surgery Memorial Sloan Kettering Cancer Center New York New York

**Keywords:** biopsy, body fluids, breast implants, fine‐needle, lymphoma, magnetic resonance imaging

## Abstract

**Background:**

To assess the incidence of benign and malignant peri‐implant fluid collections and/or masses on magnetic resonance imaging (MRI) in women with silicone implants who are being screened for silent implant rupture.

**Methods:**

The institutional review board approved this HIPAA‐compliant retrospective study and waived informed consent. Women who underwent silicone implant oncoplastic and/or cosmetic surgery and postoperative implant‐protocol MRI from 2000 to 2014 were included. Peri‐implant fluid collections and/or masses were measured volumetrically. A benign peri‐implant fluid collection and/or mass was pathologically proven or defined as showing 2 years of imaging and/or clinical stability. A malignant peri‐implant fluid collection was pathologically proven. Incidence of peri‐implant fluid collections and/or masses and positive predictive value (PPV) were calculated on a per‐patient level using proportions and exact 95% confidence intervals (CIs). Fisher's exact test was used in the analysis to test statistical significance pre‐defined as *P*‐value < 0.05.

**Results:**

A total of 1070 women with silicone implants were included (mean age, 50.7 years; range, 40.4‐53.8). Median time between reconstructive surgery and first MRI was 88.9 months (range, 0.8‐1363.3). Eighteen women (1.7%) had a peri‐implant fluid collection and/or mass: 15/18 (83.3%) had adequate follow‐up; and only 1/15 was malignant implant associated anaplastic large cell lymphoma, with a PPV of 6.7% (95% CI: 0.003‐0.0005). The median peri‐implant fluid collection size was 89 mL (range, 18‐450 mL).

**Conclusion:**

Peri‐implant fluid collections and/or masses identified at silicone implant protocol breast MR imaging are rarely seen 24 months after reconstructive surgery. Image‐guided fine‐needle aspiration with flow cytometry may be warranted to evaluate for implant‐associated lymphoma.

## Graphical abstract

Peri‐implant fluid collections and/or masses on implant protocol breast MRI are rarely seen 24 months after surgery. Image‐guided fine needle aspiration of delayed peri‐implant fluid collection may be considered, as there is a significant association with breast implant associated anaplastic large cell lymphoma.

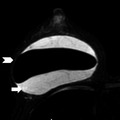

## 1 INTRODUCTION

Several case reports and large population studies^1^, ^2^, ^3^, ^4^, ^5^, ^6^, ^7^, ^8^, ^9^, ^10^ have identified a rare T‐cell lymphoma subtype in patients with breast implants, referred to as breast implant associated anaplastic large cell lymphoma (BIA ALCL). In 2011, the Food and Drug Administration (FDA) confirmed an association between breast implants and the incidence of BIA ALCL.^11^, ^12^ Since then, other organizations have confirmed this association, including the World Health Organization,^13^ the National Comprehensive Cancer Network,^14^ and the Plastic Surgery Foundation.^15^ BIA ALCL has been found to occur with both saline and silicone implants as well as oncoplastic and cosmetic surgery. It typically presents as a delayed (greater than 2 years after implant placement), symptomatic peri‐implant fluid collection; and in rare instances it is associated with a mass. Diagnosis can be made with pathological analyses including flow cytometry. However, the natural incidence of benign and malignant peri‐implant fluid collections and/or masses remains unclear, and because of this, there is uncertainty regarding the appropriate clinical management.

In regard to imaging, magnetic resonance imaging (MRI) is the most sensitive imaging modality for detecting a peri‐implant fluid collection when compared with mammogram and ultrasound.^10^ Unlike saline implants, all patients with a silicone implant are recommended by the FDA to undergo non‐contrast enhanced breast MRI 3 years after implant placement and every 2 years thereafter to exclude silent implant rupture.^16^ While non‐contrast enhanced MRI performed for silicone implant rupture is unable to screen for breast cancer due to the lack of intravenous contrast, it can readily detect peri‐implant collections and/or masses. Thus, silicone‐implant protocol MRI provides a unique opportunity to assess a subsection of the population at increased risk of BIA ALCL.

Given that BIA ALCL was only recently identified in peri‐implant fluid collections and/or masses, the management of late peri‐implant collections, that is, those identified 2 years or more post‐breast reconstruction, is unclear.^17^ It is unknown why and how frequently these collections arise and if they are an expected late appearance post‐implant placement versus an early sign of BIA ALCL. Here we evaluate the incidence of benign and malignant peri‐implant fluid collections and/or masses on MRI in women with silicone implants who are being screened for silent implant rupture.

## 2 MATERIALS AND METHODS

The institutional review board approved this Health Insurance Portability and Accountability Act‐compliant retrospective study and waived the need for informed patient consent.

### 2.1 Patients

We retrospectively searched our electronic hospital information system for women 18 years or older who met the following inclusion criteria: (a) silicone implant oncoplastic and/or cosmetic reconstructive surgery and (b) postoperative implant protocol MRI between 2000 and 2014. All women with a personal history of operable breast cancer had no clinical evidence of disease at the time of MRI. Women with locally recurrent or metastatic disease were excluded from the study. MRI studies were ordered for either (a) FDA recommended screening for silent implant rupture^16^ or (b) symptomatic implant. A total of 1070 women met the inclusion and exclusion criteria. Of 1070 patients, 923 were included in an earlier study that assessed the incidence of benign and malignant internal mammary lymph nodes on MRI in women with a history of breast cancer and silicone implant placement^18^ —and 1 patient was included in a study that evaluated the clinical and imaging findings of BIA ALCL.^19^ There was no overlap between our study and either of these publications.

### 2.2 MRI acquisition

All images were acquired with either a 1.5 or 3.0 Tesla MRI (Signa or Signa HDX; GE Medical Systems, Waukesha, WI). A dedicated four‐ or eight‐channel surface breast coil was used. Sagittal T2‐weighted images were acquired using the following parameters: repetition time (msec)/echo time (msec), 3500/102; flip angle, 90°; bandwidth, 25 kHz; field of view, 20‐24 cm; matrix, 256 × 288; number of signals acquired, 2; section thickness, 4 mm; and gap, 1 mm. Axial inversion‐recovery silicone bright water saturation images were acquired using the following parameters: 5000/34; flip angle, 90°; bandwidth, 25 kHz; field of view, 20‐24 cm; matrix, 160 × 256; number of signals acquired, 2; section thickness, 5 mm; and gap, 1 mm. Axial T2‐weighted silicone‐suppressed fat‐saturated images were acquired using the following parameters: 5000/120; flip angle, 90°; bandwidth, 32 kHz; field of view, 20‐24 cm; matrix, 320 × 224; number of signals acquired, 2; section thickness, 5 mm; and gap, 1 mm. No intravenous contrast agent was administered.

### 2.3 MRI analysis

**Figure 1:**
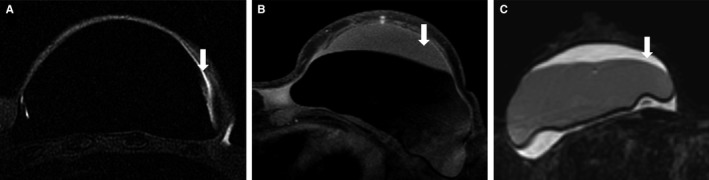
Representative cases on axial silicone‐suppressed magnetic resonance imaging (MRI) demonstrate the difference between physiologic fluid (white arrow) (A) and peri‐implant fluid collections (white arrow) (B and C)

Three radiologists (E.J.W., E.J.S., and E.A.M.) with 2, 4, and 19 years of experience, respectively, interpreted the images in consensus. The images were interpreted in random order. Trace peri‐implant fluid was considered physiologic (Figure 1). Any disagreements in interpretation was resolved by simple majority. Each case with a peri‐implant fluid collection and/or mass was volumetrically segmented on the sagittal T2‐weighted sequence and quantified by a physicist specialized in MRI (N.T.).

The MRIs were imported into an FDA‐approved image processing software, mim maestro^®^ (MIM Software, Inc Cleveland, OH 44122), currently clinically used in radiation treatment planning and various radiology applications. Peri‐implant fluid collections and/or masses were segmented on each image slice of the T2‐weighted MRI where visible. All volumetric segmentations were verified by 1 of the 3 radiologists (E.J.S.) and adjusted accordingly. The volume of peri‐implant fluid collection and/or mass was calculated by adding the volume of each voxel inside the segmented structure. The total volume (in mL) represents the volume of the peri‐implant fluid and/or mass.

### 2.4 Reference standard

Benign peri‐implant fluid collections and/or masses were pathologically proven or defined as showing at least 2 years of clinical and/or imaging stability. Malignant peri‐implant fluid collections were pathologically proven. Methods used for diagnosis were fine needle aspiration, core biopsy of a mass, or evaluation at the time of implant removal and capsulectomy. Pathologic evaluation included cytology and flow cytometry. Diagnosis of BIA ALCL required immunohistochemistry confirmation of expression of 1 or more T‐cell receptors, as well as staining positive for CD30 and negative for anaplastic lymphoma kinase‐1.

### 2.5 Statistical analysis

We examined the distribution of clinical and demographic factors by peri‐implant fluid status (Table 1). Continuous covariates were summarized as medians (ranges) and compared using the Wilcoxon‐Mann‐Whitney test. Categorical variables were summarized using frequencies and percentages. Fisher's exact test was used in the analysis to test statistical significance pre‐defined as *P*‐value <0.05. The incidence of peri‐implant fluid collections/masses and the positive predictive value (PPV) were calculated on a per‐patient level using proportions and exact 95% confidence intervals (CIs). All statistical tests were 2‐tailed and performed using SAS 9.4 (SAS Institute Inc., Cary, NC).

**Table 1:** Overall population

	n (%)
Age (median)	49
Time (months) from surgery to first MRI (mean)	47.4
History of breast cancer	
Yes	988 (92.3)
No	82 (7.7)
Peri‐implant fluid and/or mass	
Yes	18 (1.7)
No	1052 (98.3)
Lymphoma	
Yes	1 (0.09)
No	1069 (99.91)

MRI, magnetic resonance imaging.

## 3 RESULTS

In total, 1070 women with silicone implants with a mean age of 50.7 years (range, 40.4‐53.8 years) were included in the study. The mean time between reconstructive surgery and the first MRI examination was 47.4 months (range, 24.7‐53.6 months). Of the 1070 women, 807 (75.4%) had 1 MRI and 263 (24.6%) had more than 1 MRI. Of the women who had more than 1 MRI, 194 (18.1%) had 2, 58 (5.4%) had 3, 9 (0.8%) had 4, 1 (0.09%) had 5, and 1 (0.09%) had 6 (Table 1).

Of the 1070 women, 18 (1.7%) had a peri‐implant fluid collection and/or mass (Table 2). Of those with a peri‐implant fluid collection and/or mass, 15/18 (83.3%) had adequate follow‐up with 9 (50%) having pathologic diagnosis, and 6 (33.3%) having at least 2 years of clinical follow‐up and/or imaging stability. The average clinical follow‐up was 4.2 years (range, 2.1‐7.0 years). Three patients (16.7%) had inadequate follow‐up and were excluded from the PPV analysis; of the 3 patients, 2 (11.1%) died, and 1 (5.6%) was lost to follow‐up.

**Table 2:** Peri‐implant fluid and/or mass population

	n = 18 (%)
Age (mean)	57.2
Time (months) from surgery to first MRI (mean)	82.9
History of breast cancer	
Yes	14 (77.8)
No	4 (22.2)
Same side as breast cancer (n = 14)	
Yes	10 (71.4)
No	4 (28.6)
Types of fluid collection (n = 18)	
Fluid	7 (38.9)
Complex fluid	8 (44.4)
Fluid and mass	1 (5.6)
Mass	2 (11.1)
Biopsy	
Yes	9 (50)
No	9 (50)
Biopsy pathology (n = 9)	
Benign or acellular	3 (33.4)
Benign with implant rupture	1 (11.1)
Benign with inflammatory cells	2 (22.2)
Organizing hematoma	1 (11.1)
Foreign body giant cell/granulomatous reaction	1 (11.1)
Lymphoma	1 (11.1)
Adequate follow‐up or pathology	
Yes	15 (83.3)
No	3 (16.7)

MRI, magnetic resonance imaging.

**Figure 2:**
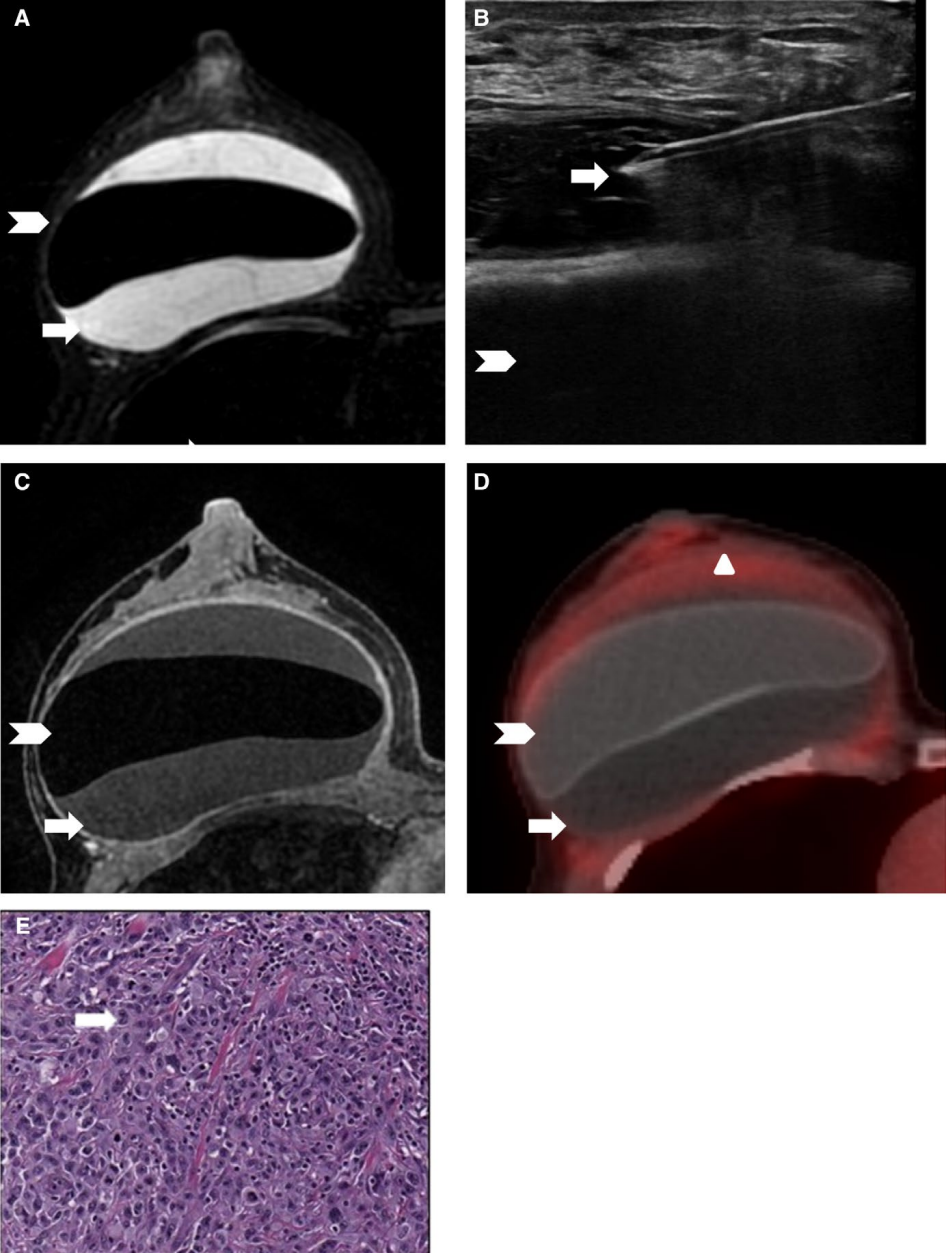
A single case of breast implant (white arrow head) associated anaplastic large cell lymphoma (BIA ALCL). Peri‐implant fluid collection (white arrow) was interpreted as suspicious and consequently the patient underwent fine needle aspiration with flow cytometry. On magnetic resonance imaging (MRI), (A) axial silicone‐suppressed MRI demonstrates a right breast peri‐implant fluid collection. (B) Targeted ultrasonography and fine needle aspiration were performed of the right peri‐implant complex fluid collection. Fluid was sent for flow cytometry which confirmed the diagnosis of BIA ALCL. (C) Breast MRI demonstrates circumferential enhancement of the fibrous capsule and no associated mass. (D) Fluorodeoxyglucose positron emission tomography–computed tomography (FDG PET‐CT), performed to define the extent of disease, demonstrates mildly increased FDG avidity of the fibrous capsule (white triangle). (E) Hematoxylin and eosin stain at 20 times magnification demonstrates large anaplastic lymphoid cells with scattered mitoses and the black arrow identifies a hallmark cell

**Figure 3:**
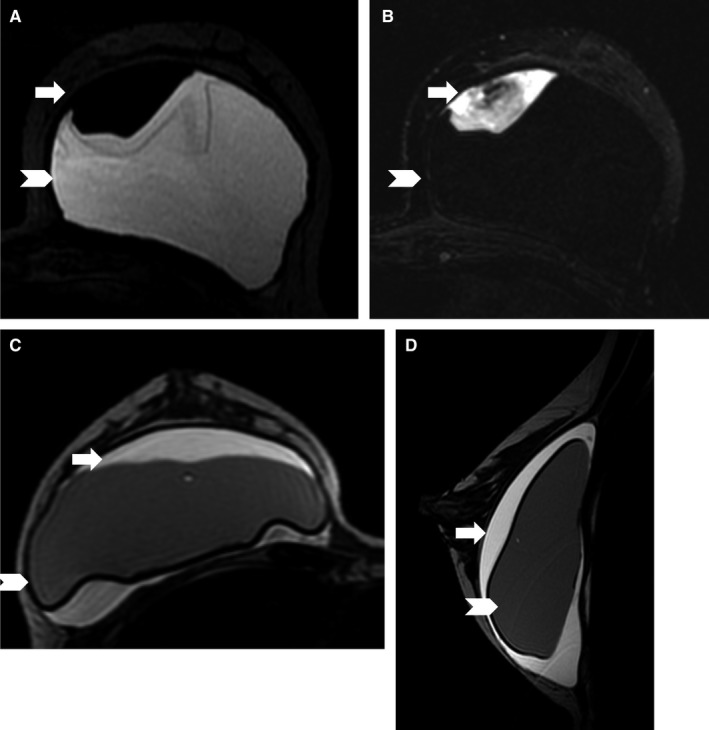
Representative cases of benign peri‐implant fluid collections. Case A: (A) Axial silicone‐bright image of the right reconstructed breast shows a hypointense peri‐implant fluid collection (white arrow) that is (B) heterogeneously hyperintense on the axial silicone‐suppressed image. This is a pathology proven chronic hematoma. Case B: Silicone‐suppressed hyperintense peri‐implant fluid collection (white arrow) in the right reconstructed breast on (C) axial and (D) sagittal images. This is a pathology proven benign acellular collection

**Figure 4:**
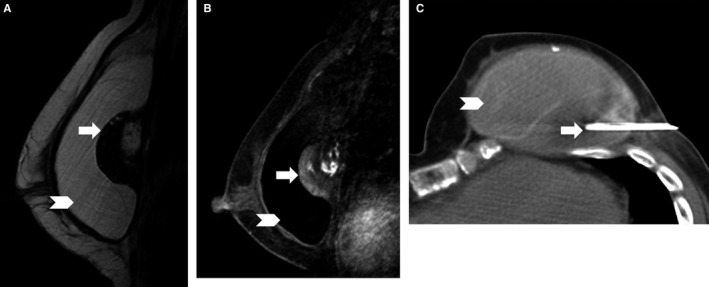
Representative case of benign peri‐implant mass. Left peri‐implant mass that is (A) heterogeneously hypointense on sagittal silicone‐bright image and (B) demonstrates heterogeneous enhancement on the post‐contrast sagittal T1‐weighted fat‐suppressed image. (C) Percutaneous computed tomography (CT)‐guided biopsy was performed and pathology was fat necrosis and necrotic fibrous tissue

The median peri‐implant fluid collection size was 89 mL (range, 18‐450 mL). Only 1 of the 15 peri‐implant fluid collections and/or masses with adequate follow‐up was malignant BIA ALCL, with a PPV of 6.7%. The patient diagnosed with BIA ALCL did not have a prior history of breast cancer but had a textured silicone implant. For the 8 benign peri‐implant fluid collections and/or masses that underwent biopsy, pathology findings were reported as follows: benign or acellular (n = 3), benign and implant rupture (n = 1), benign with inflammatory cells (n = 2), organizing hematoma (n = 1), or marked chronic inflammation and extensive foreign body giant cell/granulomatous reaction. None of the collections were infectious in etiology (Figures 2, 3, 4).

Fisher's exact test confirmed that there was a significant association between peri‐implant fluid and/or mass and the presence of lymphoma (*P* < 0.0168).

## 4 DISCUSSION

Here we found that late peri‐implant fluid and/or masses post breast reconstruction or augmentation are very rare, identified in 1.7% of patients on routine MRI performed for silicone implant rupture screening. This is in concert with prior work by Mazzocchi et al, where late peri‐implant collections were identified in 1.7% of patients after reconstruction or augmentation with textured breast implants.^20^ Other studies found lower rates of late peri‐implant seromas (0.1‐0.2%) post silicone implant reconstruction or augmentation,^21^, ^22^ which may be due to the method of detection since they did not use MRI, which would allow for detection of smaller collections not detectable on the clinical exam. We found that only 1 of the 15 delayed peri‐implant fluid collections and/or masses with adequate follow‐up in our study was malignant BIA ALCL, with a PPV of 6.7%. Our results suggest that image‐guided pathologic analysis with flow cytometry may be warranted to evaluate for implant‐associated lymphoma when delayed peri‐implant fluid collections and/or masses are diagnosed on imaging.

The current literature suggests that late peri‐implant seromas arise from friction as the implant moves within the cavity and that this friction is increased with textured rather than smooth implants.^23^ Our study demonstrates that peri‐implant collections and/or masses are more often benign and the result of an inflammatory reaction. For this reason, a working group of plastic surgeons developed a consensus statement for management of late peri‐implant collections, recommending ultrasound‐guided fine‐needle aspiration with cultures and cytologic analysis.^17^ Our study suggests that BIA ALCL should always be considered in this situation and aspirate should be sent for cytology studies with cell‐block and CD30 immunohistochemistry, and where possible, for flow cytometry and cell culture studies. Other studies suggest that textured implants may harbor more biofilm bacteria due to their greater surface area, which leads to more frequent late peri‐implant collections and may be associated with BIA ALCL.^24^, ^25^ We did not have the type of silicone implant available for our entire patient cohort.

Our study is limited for several reasons. First, we did not have adequate information on the type of silicone implant, whether textured or not textured. Second, 3 patients with peri‐implant collections and/or masses had inadequate follow‐up; thus they were excluded from the results. As such, our results may underestimate the prevalence of BIA ALCL. In addition, there was an inherent selection bias in that we evaluated only peri‐implant collections associated with silicone implants and 92.3% of our patients had a past medical history of breast cancer.

In conclusion, late peri‐implant fluid collections and/or masses are rarely identified at silicone implant protocol breast MRI 24 months or more after reconstructive surgery. If a late peri‐implant fluid collection and/or mass is detected, ultrasound‐guided fine‐needle aspiration with CD30 immunohistochemistry and cell‐block cytology should be considered to exclude BIA ALCL.

## CONFLICT OF INTEREST

None.

## AUTHORS’ CONTRIBUTIONS

Elizabeth J Sutton MD: Conceptualization, data curation, formal analysis, funding acquisition, investigation, methodology, project administration, resources, software, supervision, validation, visualization, writing—original draft, and writing—review and editing. Brittany Z Dashevsky MD DPhil: Data curation, investigation, methodology, project administration, writing—original draft, and writing—review and editing. Elizabeth J. Watson MD: Data curation, investigation, methodology, project administration and writing—review and editing. Neelam Tyagi PhD: Data curation, formal analysis, software and writing—review and editing. Blanca Bernard‐Davila MPH MS: Formal analysis, software and writing—review and editing. Danny Martinez BSC MSc: Data curation, formal analysis, software and writing—review and editing. Ahmet Dogan MD PhD: Data curation, formal analysis, supervision and writing—review and editing. Steven M Horwitz MD: Conceptualization, formal analysis, supervision and writing—review and editing. Peter G Cordeiro MD: Data curation, formal analysis, supervision and writing—review and editing. Elizabeth A. Morris MD: Conceptualization, formal analysis, supervision and writing—review and editing.
